# Impacts of Microbial based Therapy on Growth Performance, Intestinal Health, Carcass Traits and Economic Efficiency of *Clostridium perfringens*‐Infected Cobb and Arbor Acres Broilers

**DOI:** 10.1002/vms3.412

**Published:** 2021-03-15

**Authors:** Eman A. Sallam, Liza S. Mohammed, Sawsan S. Elbasuni, Aya E. Azam, Mohamed Mohamed Soliman

**Affiliations:** ^1^ Animal and Poultry Production Animal Wealth Development Department Faculty of Veterinary Medicine Benha University Benha Egypt; ^2^ Veterinary Economics and Farm Management Animal Wealth Development Department Faculty of Veterinary Medicine Benha University Benha Egypt; ^3^ Avian and Rabbit diseases Department Faculty of Veterinary Medicine Benha University Benha Egypt; ^4^ Animal Hygiene and Veterinary Management Faculty of Veterinary Medicine Benha University Benha Egypt; ^5^ Clinical Laboratory Sciences Department Turabah University College Taif University Taif Saudi Arabia

**Keywords:** broilers, carcass traits, growth performance, intestinal health, microbial‐based therapy

## Abstract

The poultry farms need a safe and effective alternative for antibiotics that can counteract the negative impacts of necrotic enteritis (NE), which causes severe mortalities and economic losses. The current study was aimed to examine the influence of antibiotic (Flagymox) and the microbial‐based administration on carcass traits in *Clostridium* *(C*.*)* *perfringens*‐infected Cobb and Arbor Acres broilers. A total number of 360 Cobb and Arbor Acres broiler chicks (180 numbers per breed) were allocated to four groups; negative control group (without any treatments); positive control group (administration of *C*. *perfringens* at the rate of 1 × 10^9^cfu/bird via crop gavage twice daily from day 16 to 18 post‐hatch); *C*. *perfringens* challenge plus antibiotic (Flagymox^®^) group, and *Clostridium* *perfringens* challenge plus microbial‐based treatment (Big‐lactoα^®^) group. The results indicated that the Flagymox and Big‐lactoα treated Cobb breed group achieved a significant increase in their body weight (BW) than the positive control group at the third week post‐infection. Also, the Arbor Acres breed gained significantly higher weight compared to the Cobb breed at the third week. Total weight gain (TWG) from 0 to the fifth week in the Cobb and Arbor Acres breeds were higher in the groups treated with Flagymox and Big‐lactoα compared to the birds challenged with *C*. *perfringens* without any treatment, thus, increasing the total return (TR) in the treated groups. Economic efficiency showed no significant differences (*p* < .05) between the treatment groups of both the breeds. Although the treatment cost of Flagymox is higher than the microbial‐based treatment (0.86 versus 0.35 LE), there were no mortalities reported in the microbial‐based groups in both the breeds resulting in significantly low losses compared to the Flagymox treated groups. The groups treated with the microbial‐based products in both breeds were superior in dressing percentage (75.16 and 77.06% for Cobb and Arbor Acres, respectively) compared to that of the other groups. In conclusion, microbial‐based therapy improved the growth rate, carcass traits, survival rate, and economic efficiency in necrotic enteritis induced in Cobb and Arbor Acres broilers.

## Graphical abstract

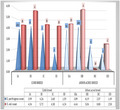

## 1 INTRODUCTION

Necrotic enteritis (NE) is one of the most widespread enteric diseases found in broilers. It has a major impact on the cost‐efficiency of meat production due to the increased mortality, veterinary intervention, and treatment costs (Whelan et al., 2019). The NE‐related global economic losses in the poultry industry are estimated to be over 6 billion US dollars annually (Wade et al., 2015). This disease causes a multi‐factorial gut health issue that depends on interactions between several factors, such as host, infectivity, nutritional and managerial factors. The presence of *Clostridium* *(C*.*)* *perfringens*, usually found in the healthy chicken intestines, with some of the additional and predisposing factors that modify the intestinal ecosystem allowing *C*. *perfringens* to overgrow, eliciting the clinical signs and lesions of NE. Studies (Jang et al., 2013; Tsiouris, 2016) have reported susceptibility differences in Cobb, Ross, and Hubbard broilers in developing NE. Cobb chicken fed with a high protein diet was the most susceptible breed to orally infected *Eimeria* *maxima* and viable *C*. *perfringens* (Jang et al., 2013; Tsiouris, 2016). Moreover, Kim et al. (Kim et al., 2014) documented the disparate NE susceptibility in two inbred Ross chicken lines having variations in transcriptional profiles of host–pathogen response against the disease with differentially regulated immune genes (Osman & Elhariri, 2013). Several strategies were applied to reduce the incidence of NE and maintain the profitability of broiler production (Skinner et al., 2010). The usage of antimicrobial drugs, anticoccidials, and/or antibiotics in feed and/or drinking water are increased nowadays to enhance the growth and feed efficiency through their antibacterial activity against C. *perfringens* (Williams, 2005). A sense of urgency to find an alternative approach has overcome the strong challenges in the global poultry industry as there is an increase in the incidence of NE in Europe and the USA because of the European Union ban in 2006 that restricts the usage of all antibiotics in all animal feed (M’Sadeq et al., 2015). This is also accompanied by the high acquisition resistance of C. *perfringens* isolates from broiler chickens against multiple antibiotics (Osman & Elhariri, 2013).

Live microorganisms, prebiotics, plants and their extracts, organic acids, enzymes, lysozyme, yeast extract and antimicrobial peptides are all employed in the intervention strategies used for C. *perfringens* induced NE in broiler chickens, which could support a one health approach (Caly et al., 2015). As known, bacteriophages are highly species‐specific viruses, and predators of bacteria and hence are widely used to treat bacterial infections in humans, animals and poultry (Caly et al., 2015; Miller et al., 2010; Seal et al., 2018). Some papers have documented the efficiency of using feed supplementation of six bacteriophages to reduce the mortality and improve weight gain (WG) and feed conversion rate (FCR) in the NE challenged chicken. Moreover, probiotics are the live yeast or bacteria used in the feed to ameliorate the adverse effects of the *C*. *perfringens* infected broilers on their performance and intestinal health by reducing the risk of opportunistic and pathogenic bacterial infection (Hussein, Ahmed, Abudabos, Aljumaah, et al., 2020; Khalique et al., 2020; Whelan et al., 2019).

Therefore, this study was aimed to determine and outline the influence of antibiotics and microbial‐based administration (Flagymox and Big‐lactoα) on the growth performance traits, intestinal health measurement, carcass traits and the measurement of economic efficiency of *C*. *perfringens* infected Cobb and Arbor Acres broilers.

## 2 MATERIAL AND METHODS

### 2.1 Birds and the treatments

A total of 360, i.e., 180 Cobb broiler and 180 Arbor Acres broiler chicks, were used for an experiment period of 35 days. The chicks were maintained in well‐ventilated litter floor rooms and stocked at a density of 10 birds/m^2^. All birds were subjected to the same managerial, hygienic and housing conditions and were vaccinated against Newcastle disease and infectious bursal disease. Broiler breeds were randomly distributed into four groups, and each group contained three replicates (15 birds per replicate) with both breeds assigned to one of the following treatments (Table 1):

**Table 1:** Experimental design and different treatments

Breed	Experiment Groups		Challenge with *Cl*. *Perfringens*			Treatments^a^, ^b^		
	Name	Code	Time	Dose	Route	Type	Time	Route
Cobb	Control negative	IA	—	—	—	—	—	—
	Control positive	IB	day 16 to 18 post‐hatch	1 × 10^9^ cfu /bird	crop gavage	—	—	—
	Antibiotic treatment^a^	IC				Flagymox®	once daily /17−19 day Post‐hatch	D.W.
	Microbial based treatment^b^	ID				Big‐lactoα^®^		
Arbor Acres	Control negative	IIA	—	—	—	—	—	—
	Control positive	IIB	day 16 to 18 post‐hatch	1 × 10^9^ cfu /bird	crop gavage	—	—	—
	Antibiotic treatment^a^	IIC				Flagymox^®^	once daily /17−19 day Post‐hatch	D.W.
	Microbial based treatment^b^	IID				Big‐lactoα^®^		

^a^ **Flagymox^®^: it is** manufactured by ATCO Pharma Trading Co., Egypt and each 100 mg of product contain 10 mg amoxicillin plus 20 mg metronidazole.

^b^ **Big‐lactoα** ^®^: it is provided by Biogenoci Co ltd. Its ingredients are Lactobacillus acidophilus (5x10^8^CFU/ vial) and bacteriophage (>1x10^8^ PFU/g).

Negative control groups (IA and IIA), no challenge and no treatment.

Positive control groups (IB and IIB), challenged with *C*. *perfringens*.

*C*. *perfringens* treated with antibiotic Flagymox groups (IC and IIC).

*C*. *perfringens* challenged and treated with a microbial‐based product groups (ID and IID).

The *C*. *perfringens* challenge model was performed based on previously published studies (McReynolds et al., 2004; Prescott et al., 2016; Shojadoost et al., 2012). All challenged groups received *C*. *perfringens* at the rate of 1 × 10^9^cfu/bird via crop gavage twice daily from day 16 to 18 post‐hatch. *C*. *perfringens* was obtained from Department of Bacteriology and Immunology, Faculty of Veterinary Medicine, Benha University.

### 2.2 Preparation of microbial‐based treatment (Big‐lactoα)

Freeze‐dried anaerobic *Probiotic* *and* *Bacteriophage* *combination* presented in a vial was used for this experiment. The active ingredients of BIG‐LACTOα were Probiotics (*Lactobacillus* *acidophilus*: >5x10^8^ CFU per vial) and Bacteriophages (used against several pathogens, >1x10^8^ PFU per gram) along with prebiotics and an edible colouring agent. Big‐lactoα^®^: was purchased from Biogenoci Co ltd, Osaka, Japan).

### 2.3 Measurement of the Growth Performance

During the experiment (days 0–35), the growth performance of the broiler chicks was evaluated by recording their daily and weekly feed intake (FI). The live body weight (BW) was monitored weekly to calculate body weight gain (WG). The feed conversion ratio (FCR) was calculated as reported by Elbayoumi et al., 2014. Also, dead birds in each group were recorded daily to calculate the mortality rate as described by Miller et al., 2010, and European Broiler Index (EBI) was estimated as described by Mathis et al., 2016.

### 2.4 Assessment of the intestinal health

On day 21 of post‐hatch, five birds were randomly selected from each group and humanely sacrificed by cervical dislocation. The intestinal tissues were then collected for the following parameters:

### 2.5 Bacterial Quantification

To estimate the counts of *E*. *coli* and C. *perfringens*, a piece of the jejunum (15 cm) from the sacrificed birds were placed in 10 ml of anaerobic fluid thioglycollate broth (Oxoid microbiology product, Thermo Scientific Co, USA) under septic conditions for 30 s. 0.5‐mL of this sample was placed into 4.5 ml of neutral physiological buffer saline to perform 10‐fold serial dilutions, where each dilution was plated onto the MacConkey agar and incubated at 37°C for 24 hr. Also, another 0.5‐mL of the sample was used for 10‐fold serial dilutions in fluid thioglycollate broth, and 1 ml of each dilution was poured on tryptose sulfite cycloserine agar plates and further incubated anaerobically at 37°C for 24 hr. The inoculated plates that had more than 30 or less than 300 colonies of *E*. *coli* and *C*. *perfringens* with typical morphology were recorded (McReynolds et al., 2004).

### 2.6 Lesion scoring and Morphometric analysis

To evaluate the gross lesions of necrotic enteritis, 10 cm of jejunum section from the sacrificed birds were examined macroscopically and scored from 0 to 4 using previously documented criteria (Prescott, 1979). Then, the jejunum samples were fixed in phosphate‐buffered formalin for at least 24h and embedded in paraffin. Sections of 5 mm were cut and stained with haematoxylin and eosin (Bancroft & Gamble, 2008). The stained sections were microscopically examined for histopathological changes such as epithelial cell necrosis, inflammatory cells, fibrin exudate in the gut lumen, and epithelial hyperplasia, which were recorded and scored between 0 to 4 based on the published papers (Dahiya et al., 2007). Also, the intestinal morphometric variables, including the villi length, crypt depth, villi depth and the extent of tissue injury, were measured using the Image capture. Furthermore, the analysis system (Langhout, 1998; Wealleans et al., 2017) was used to calculate the villi length and crypt depth ratio.

### 2.7 Total RNA extraction, cDNA synthesis and quantitative real‐time PCR (qRT‐PCR)

To achieve a better understanding of the effects of NE on jejunal cytokine and chemokine production (IL‐6 and IL‐8), the effects of antibiotics and microbial‐based product administration were examined for their expression in the jejunum. The expressions of jejunal IL‐6 and IL‐8 genes were analysed by real‐time PCR using sense and antisense primers. Intestinal tissue (jejunum region) was cut longitudinally, washed with saline, blotted on a filter paper and maintained at −80°C until RNA extraction. Total RNA from jejunum was isolated using TRIzol (Invitrogen™, Thermo fisher scientific) reagent as described according to the manufacturer's protocol. The concentration and purity of total RNA were estimated by measuring the optical density at 260 and 280 nm. 5 µg of total RNA from each sample was reverse transcribed into cDNA using a High Capacity cDNA Reverse Transcription Kit (Applied Biosystems, CA, USA) according to the manufacturer's instructions.

For cDNA quantification, the RT‐PCR assay was performed with the 7,500 Fast Real‐time PCR detection system (Applied Biosystems) as per the optimized PCR protocols using the Maxima SYBR Green qPCR Master Mix (2X) kit (Thermo Fisher). PCR was performed in a 20‐µL reaction volume containing 10µl of Maxima SYBR Green qPCR Master Mix, 1 µM of each sense and antisense primers, 1 µl of 1µg/µl cDNA and nuclease‐free water to make up the final volume of 20 µl. Primer sets were as follows: IL‐6 (Gene Bank ID: AJ309540), sense (5′‐CAAGGTGACGGAGGAGGAC‐3′) and antisense (5′‐TGGCGAGGAGGGATTTCT‐3′); IL‐8 (Gene Bank ID: AJ009800), sense (5′‐GGCTTGCTAGGGGAAATGA‐3′) and antisense (5′‐AGCTGACTCTGACTAGGAAACTGT‐3′); and GAPDH as a housekeeping gene (Gene Bank ID: K01458), sense (5′‐GGTGGTGCTAAGCGTGTTAT‐3′) and antisense (5′‐ACCTCTGTCATCTCTCCACA‐3′).

The real‐time PCR cycling program was as follows: 95°C for 10 min (holding stage), 95°C for 15 s for 40 cycles (denaturation stage), followed by 60°C for 1 min (annealing and extension stage). The changes in the gene expression were calculated by the 2^−ΔΔCt^ method using the cycle threshold (Ct) values, where ^Δ^Ct indicated the Ct changes in target genes compared to a reference (housekeeping) gene (GAPDH) (Schmittgen & Livak, 2008).

### 2.8 Measurement of carcass quality

At the end of the growing period (day 35), five birds were randomly collected from each group and left for 12 hr to fast. Each bird's carcass was weighed before the slaughter and after complete dressing (removal of the feather, head, neck, shanks, feet and viscera). The dressing percentage was calculated according to the method of Brake et al., 1995. All the internal organs (Heart, gizzard, liver without the gall bladder, spleen, thymus, bursa, abdominal fat and gizzard fat) were also weighed immediately after the sacrifice, and the gizzard was weighed after the removal of its content.

### 2.9 The measurement of economic efficiency

The economic efficiency at the end of the experiment (day 35) was evaluated using total costs (TC), which included total variable cost (TVC) and total fixed cost (TFC). TVC included feed consumption, total veterinary management (TVM), labour, chick price, water, electricity and litter costs and was estimated in Egyptian pound LE (1 USD ≈ 17.6 LE) for every bird in all the groups during the period of the experiment. TFC was estimated at 1.61 LE/bird, which included depreciation of buildings and equipment (Amarapurkar et al., 2014). Total returns (TR) equaled the summation of returns obtained from selling bird and litter, where net profit was TR–TC (Abudabos et al., 2018), economic efficiency (EE)= net profit/TC, relative economic efficiency (REE)= EE (tested group)/EE (control group) X 100 according to Eman et al. (Kamel et al., 2020), and losses due to mortality = *N* (no. of dead chickens)×[VC (value of a day‐old chick)+CCF (cost of cumulative feed consumed by a single bird X no. of dead birds)+total rearing cost (Györke et al., 2016).

### 2.10 Statistical analysis

Data obtained in the current study were statistically analysed for analysis of variance (ANOVA) (Significance at *p* ≤ .05), and the breed effect was analysed by independent sample T‐test using SPSS/PC^+^ “version 16 (Guide, 2002). The results were represented as means ± standard error (SE). Differences between the groups were analysed using Duncan's multiple Post Hoc tests, and Pearson's correlation was used to analyse the association between the macroscopic lesion scores of the jejunum and its morphometric variables.

## 3 RESULTS

### 3.1 The effect of tested antibiotics and microbial‐based administration on the body weight changes (BW) and feed intake (FI) of experimentally infected Cobb and Arbor Acres broilers with *C. perfringens*

Table 2 represents the changes in the body weight of the birds at the third week of post‐infection. The treatment groups (IC & ID) in the Cobb breed achieved a significant increase in the body weight (BW) (822.11 g and 839.57 g for antibiotics and microbial‐based products, respectively) compared to the positive control group (759.01 g).

**Table 2:** Effect of antibiotic and microbial based administration in Cobb and Arbor acres broilers experimentally infected with *Cl*. *perfringens* on body weight changes (BW) and feed intake (FI)

Items	Cobb Breed					Arbor acres Breed				
	IA	IB	IC	ID	Overall mean	IIA	IIB	IIC	IID	Overall mean
BW 1st week	167.44^a^ ± 2.81	165.78^a^ ± 3.31	171.55^a^ ± 0.22	166.99^a^ ± 4.56	167.94^A^ ± 1.49	166.89^a^ ± 6.44	159.78^a^ ± 4.41	163.03^a^ ± 5.27	167.55^a^ ± 3.95	164.31^A^ ± 2.37
BW 2nd week	425.56^b^ ± 4.81	416.84^b^ ± 8.25	435.80^ab^ ± 11.42	457.78^a^ ± 4.43	433.99^A^ ± 5.67	418.33^b^ ± 10.09	413.49^b^ ± 11.53	414.16^b^ ± 14.50	433.17^ab^ ± 10.34	419.79^A^ ± 5.55
BW 3rd week	777.02^ab^ ± 9.80	759.01^b^ ± 7.45	822.11^ab^ ± 36.75	839.57^a^ ± 20.83	799.43^A^ ± 13.60	846.07^a^ ± 7.54	819.80^ab^ ± 21.67	834.82^ab^ ± 37.47	800.03^ab^ ± 21.77	825.18^A^ ± 11.68
BW 4th week	1,072.85^b^ ± 58.27	1,066.24^b^ ± 16.64	1,313.94^a^ ± 73.39	1,168.02^ab^ ± 14.97	1,155.26^A^ ± 36.50	1,235.90^a^ ± 28.73	1,170.25^ab^ ± 50.22	1,299.58^a^ ± 35.59	1,198.37^ab^ ± 41.98	1,226.03^A^ ± 22.43
BW 5th week	1546.80^a^ ± 90.27	1,485.27^a^ ± 84.52	1711.82^a^ ± 144.17	1599.86^a^ ± 62.56	1585.94^A^ ± 49.47	1637.38^a^ ± 46.23	1609.59^a^ ± 10.36	1641.33^a^ ± 70.01	1632.67^a^ ± 113.05	1,630.24^A^ ± 0.32
FI 1st week	153.22^a^ ± 3.53	140.33^ab^ ± 2.17	154.11^a^ ± 4.33	145.19^ab^ ± 5.82	148.21^A^ ± 2.48	146.33^ab^ ± 0.88	136.28^b^ ± 4.54	154.06^a^ ± 8.07	148.44^ab^ ± 3.53	146.27^A^ ± 2.87
FI 2nd week	325.44^ab^ ± 2.95	317.67^bc^ ± 2.14	339.22^a^ ± 8.81	339.64^a^ ± 5.16	330.49^A^ ± 3.65	319.67^bc^ ± 4.51	302.15^c^ ± 2.55	328.74^ab^ ± 10.89	320.33^bc^ ± 0.19	317.72^B^ ± 3.89
FI 3rd week	575.87^ab^ ± 5.93	541.33^b^ ± 5.57	583.44^ab^ ± 15.53	603.07^a^ ± 7.86	575.93^A^ ± 7.88	588.53^a^ ± 7.29	581.60^ab^ ± 16.41	593.72^a^ ± 27.19	616.53^a^ ± 10.97	595.10^A^ ± 8.33
FI 4th week	1,130.95^a^ ± 103.28	985.73^a^ ± 46.83	1,144.18^a^ ± 58.75	1,070.23^a^ ± 61.27	1,082.77^A^ ± 35.61	1,047.08^a^ ± 102.97	1,135.59^a^ ± 16.30	1,161.62^a^ ± 38.40	1,045.38^a^ ± 45.59	1,097.418^A^ ± 30.02
FI 5th week	1,452.67^a^ ± 7.54	1,475.13^a^ ± 81.51	1,446.00^a^ ± 12.86	1,417.33^a^ ± 16.34	1,447.78^A^ ± 19.05	1,424.78^a^ ± 13.80	1,459.27^a^ ± 79.45	1,389.70^a^ ± 20.38	1,380.57^a^ ± 84.87	1,413.58^A^ ± 27.02
TFI	3,638.15^a^ ± 115.70	3,460.20^a^ ± 30.41	3,666.95^a^ ± 35.66	3,575.46^a^ ± 54.39	3,585.187 ^A^ ± 37.63	3,526.40^a^ ± 103.86	3,614.89^a^ ± 57.86	3,627.84^a^ ± 69.98	3,511.26^a^ ± 137.32	3,570.097^A^ ± 44.34

Note

Small letters are significantly different between treated and untreated groups (*p* ≤ .05), while Capital letters significantly differ among different breeds.

At the fourth week, the antibiotic‐treated groups of both breeds (IC & IIC) achieved the highest values of BW (1,313.94 g and 1,299.58 g for Cobb and Arbor Acres, respectively) while the lowest value (1,066.24 g) was recorded in the positive control of the Cobb breed (IB). However, at the fifth week, though no significant differences were observed in the BW among the groups, compared to the positive control group (IB and IIB) in both the breeds, a numerical increase was observed in the weights of the bird groups treated with antibiotics and microbial‐based products (IC, ID & IIC, IID). Concerning the effect of the breed in the first and second weeks, the Cobb breed outperformed the Arbor Acres breed in the BW, but in the third, fourth and fifth weeks, the Arbor Acrer breed exceeded the Cobb breed's BW. At the third week, there was a significant difference in the feed intake (FI) between the treated (IC, ID & IIC, IID) and untreated groups (IA, IB & IIA, IIB). A higher value of FI was recorded in the groups treated with antibiotics, microbial‐based products and the negative control (IA, IC, ID & IA, IIC, IID) compared to the positive control groups (IB & IIB) of both the breeds. The FI in the fourth and fifth weeks, along with the total feed intake (TFI), showed non‐significant differences among different groups.

### 3.2 The effect of antibiotics and microbial‐based administration on body weight gain changes (WG) and feed conversion ratio (FCR) in experimentally infected Cobb and Arbor Acres broilers infected with *C. perfringens*

The results represented in Table 3 showed that WG at the second week of pre‐infection was significantly higher in the treated microbial‐based groups (ID & IID) compared to other groups of both breeds (290.79 g and 265.62 g for Cobb and Arbor Acres, respectively). Regarding weight gain (WG) of Cobb breed at third week post‐infection, treated groups with antibiotics and microbial‐based products (IC & ID) showed a high WG (386.31 g and 381.79 g, respectively) compared to the negative and positive control groups (351.47 g and 342.17 g, respectively). Due to the effect of the breed on WG, the Arbor Acres breed (405.39 g) showed a significantly higher weight gain than the Cobb breed (365.44 g). The WG at the fourth week showed a significant difference among different groups. In both breeds, the treated groups (IC, ID & IIC, IID) showed an increase in WG compared to the negative and positive control groups (IA, IB & IIA, IIB).

**Table 3:** Effect of antibiotic and microbial based administration in Cobb and Arbor acres broilers experimentally infected with *Cl*. *perfringens* on body weight gain changes (WG) and feed conversion ratio (FCR)

Items	Cobb Breed					Arbor acres Breed				
	IA	IB	IC	ID	Overall mean	IIA	IIB	IIC	IID	Overall mean
WG 1st week	117.56^a^ ± 1.93	117.44^a^ ± 3.97	122.00^a^ ± 0.39	117.88^a^ ± 4.38	118.72^A^ ± 1.45	120.89^a^ ± 6.45	115.22^a^ ± 3.85	117.36^a^ ± 5.28	122.11^a^ ± 3.89	118.89^A^ ± 2.28
WG 2nd week	258.11^b^ ± 2.06	251.06^b^ ± 5.02	264.24^b^ ± 11.56	290.79^a^ ± 4.16	266.05^A^ ± 5.36	251.45^b^ ± 3.80	253.71^b^ ± 7.49	251.13^b^ ± 9.44	265.62^b^ ± 6.58	255.47^A^ ± 5.53
WG 3rd week	351.47^cd^ ± 10.02	342.17^d^ ± 1.64	386.31^abcd^ ± 25.66	381.79^abcd^ ± 24.41	365.44^B^ ± 9.72	427.73^a^ ± 6.68	406.31^abc^ ± 19.70	420.66^ab^ ± 25.75	366.86^bcd^ ± 12.26	405.39^A^ ± 10.35
WG 4th week	295.82^c^ ± 57.91	307.23^c^ ± 14.75	491.82^a^ ± 36.64	328.45^c^ ± 20.55	355.83^A^ ± 28.56	389.83^abc^ ± 34.94	350.45^bc^ ± 64.66	464.76^ab^ ± 17.68	398.34^abc^ ± 21.90	400.85^A^ ± 20.86
WG 5th week	473.95^a^ ± 39.73	419.03^a^ ± 97.24	397.88^a^ ± 70.87	431.84^a^ ± 48.90	430.68^A^ ± 30.14	401.48^a^ ± 33.51	439.34^a^ ± 47.57	341.75^a^ ± 36.95	434.30^a^ ± 71.10	404.22^A^ ± 24.15
TWG	1,496.91^a^ ± 89.35	1,436.94^a^ ± 84.61	1662.27^a^ ± 144.62	1,550.75^a^ ± 62.73	1536.72^A^ ± 49.42	1591.38^a^ ± 46.31	1565.03^a^ ± 11.43	1595.66^a^ ± 70.02	1587.23^a^ ± 113.07	1584.83^A^ ± 30.33
FCR 1st week	1.30^a^ ± 0.01	1.20^a^ ± 0.03	1.26^a^ ± 0.03	1.23^a^ ± 0.04	1.24^A^ ± 0.02	1.22^a^ ± 0.06	1.18^a^ ± 0.03	1.32^a^ ± 0.08	1.22^a^ ± 0.03	1.23^A^ ± 0.03
FCR 2nd week	1.26^ab^ ± 0.00	1.27^ab^ ± 0.02	1.29^ab^ ± 0.04	1.17^b^ ± 0.02	1.25 ± 0.02	1.27^ab^ ± 0.04	1.19^ab^ ± 0.03	1.31^a^ ± 0.08	1.21^ab^ ± 0.03	1.25 ± 0.03
FCR 3rd week	1.64^a^ ± 0.06	1.58^ab^ ± 0.02	1.52^abc^ ± 0.07	1.59^ab^ ± 0.10	1.58^A^ ± 0.03	1.38^c^ ± 0.03	1.43^bc^ ± 0.04	1.42^bc^ ± 0.07	1.68^a^ ± 0.05	1.48^A^ ± 0.04
FCR 4th week	3.98^a^ ± 0.43	3.21^ab^ ± 0.07	2.36^b^ ± 0.23	3.31^ab^ ± 0.40	3.21^A^ ± 0.22	2.69^b^ ± 0.09	3.50^ab^ ± 0.71	2.51^b^ ± 0.14	2.63^b^ ± 0.10	2.83^A^ ± 0.20
FCR 5th week	3.11^a^ ± 0.26	3.90^a^ ± 0.87	3.85^a^ ± 0.61	3.37^a^ ± 0.40	3.56^A^ ± 0.27	3.59^a^ ± 0.26	3.43^a^ ± 0.52	4.16^a^ ± 0.43	3.31^a^ ± 0.40	3.62^A^ ± 0.20
TFCR	2.44^a^ ± 0.10	2.42^a^ ± 0.13	2.24^a^ ± 0.19	2.32^a^ ± 0.13	2.36^A^ ± 0.07	2.22^a^ ± 0.06	2.31^a^ ± 0.04	2.28^a^ ± 0.06	2.22^a^ ± 0.08	2.26^A^ ± 0.03

Note

Small letters are significantly different between treated and untreated groups (*p* ≤ .05), while Capital letters significantly differ among breeds .BWG) was calculated by subtracting the body weight between two successive weights in different weeks of experiment, TWG (final body weight‐ initial body weight) and feed conversion rate (FCR)= feed intake (FI)/ body weight gain (BWG).

There was a non‐significant difference in the WG between different groups in the fifth week for both the breeds. As for the total weight gain (TWG) from zero to the fifth week in the Cobb breed, a numerical increase was observed in the groups treated with antibiotics and microbial‐based products (IC, 1662.27 g & 1,550.75 g for ID group, respectively) compared to the untreated groups (IA & IB), which also showed a low TWG in the positively challenged group (IB) (1,436.94 g). The FCR at the fourth week post‐infection was significantly different between the groups. The final feed conversion from zero to fifth week showed non‐significant differences among different groups and different breeds.

### 3.3 The effect of antibiotics and microbial‐based administration on European broiler index (EBI) in experimentally infected Cobb and Arbor Acres broilers with *C. perfringens*

**Figure 1:**
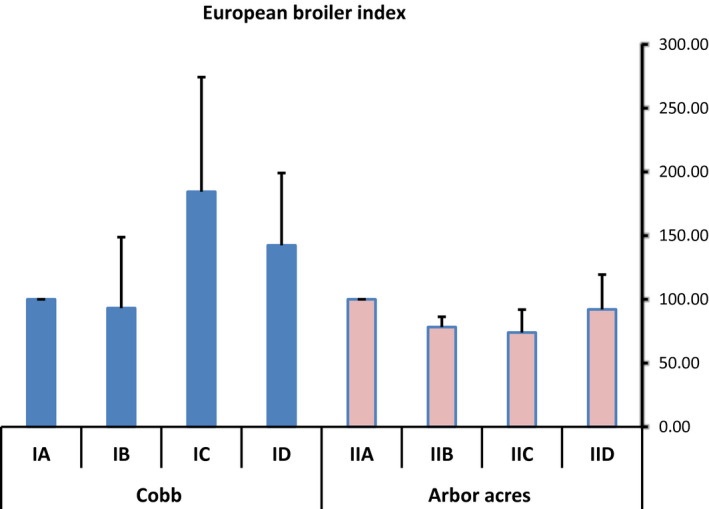
The effect of antibiotics and microbial‐based administration on the European broiler index in Cobb and Arbor Acres broilers experimentally infected with *C*. *perfringens*

The groups treated with antibiotics and microbial‐based products (IC, ID & IIC, IID) had higher EBI values when compared to the positive control groups (IB & IIB) in Cobb and Arbor Acres breed. Also, a non‐significant difference (*p* < .05) was observed in the EBI values between both the breeds, as shown in Figure 1.

### 3.4 The effect of antibiotics and microbial‐based administration on post‐infection mortality and livability percentage of experimentally infected Cobb and Arbor Acres broilers with *C. perfringens*

**Figure 2:**
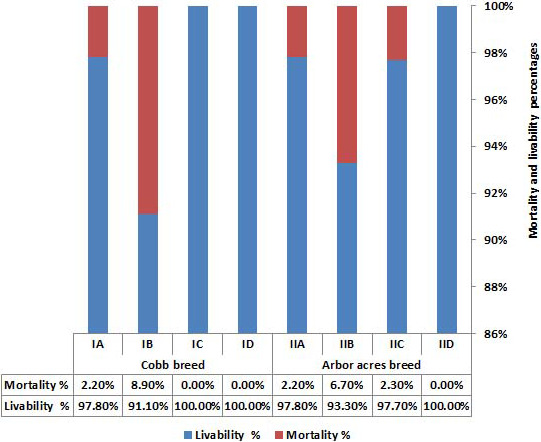
The effect of antibiotics and microbial‐based administration on post‐infection mortality and livability percentage in Cobb and Arbor Acres broilers experimentally infected with *C*. *perfringens*

Regarding the mortality percentage, a difference was observed between the treated and untreated groups of NE infected broilers (Figure 2). The highest mortality percentages were 8.9% in the NE infected Cobb birds followed by 6.7% in the infected Arbor Acres chicks compared to the positive control groups (IB & IIB). The treatment with antibiotics or microbial‐based products on the breeds infected with *C*. *perfringens* showed a decrease in the mortalities compared to the positive control groups (IB & IIB). Importantly, no mortalities were recorded in the NE infected Cobb and Arbor Acres broilers treated with microbial‐based products (ID & IID).

### 3.5 The effect of antibiotics and microbial‐based therapy on the counts of *C. perfringens* and *E. coli* in the jejunum of experimentally infected Cobb and Arbor Acres broilers with *C. perfringens*

**Figure 3:**
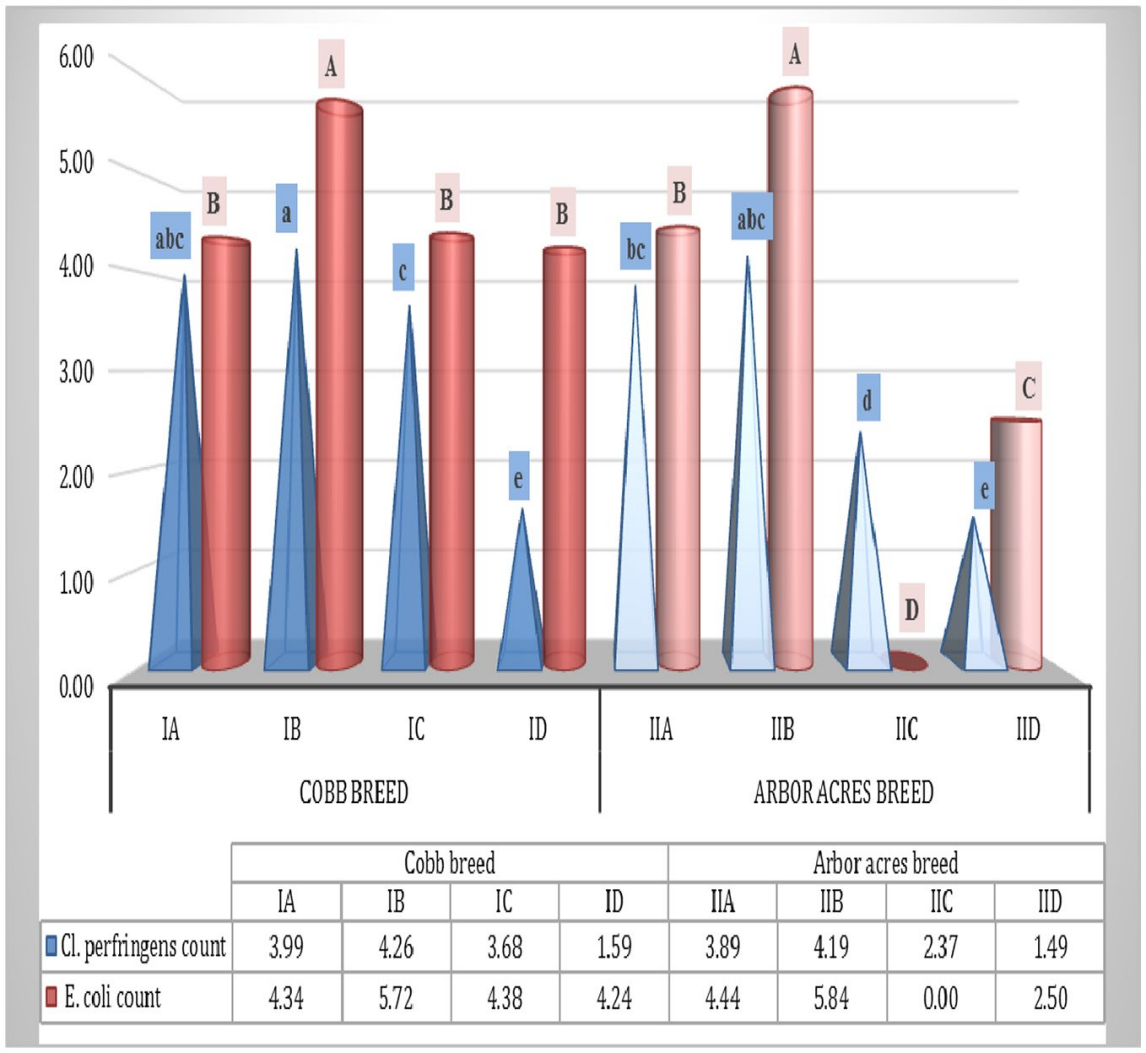
The effect of antibiotics and the microbial‐based therapy on the counts of *C*. *perfringens* and E. coli in the jejunum of experimentally infected Cobb and Arbor Acres broilers with *C*. *perfringens*. The values are presented as bars with different letters (a–e) and show significantly different mean values of *C*. *perfringens* count (log10 cfu/mL) between treated and untreated groups (*p* < .05). A significant difference (*p* < .05) was observed in the mean values of the E.coli count (log10 cfu/mL) between different experimental groups, which is illustrated as bars with different letters (A‐D)

The diversity of *C*. *perfringens* and *E*. *coli* in the jejunum of the experimental birds are presented in Figure 3 and show high values of 4.26 and 5.72 CFU/mL for IB and 4.19 and 5.84 CFU/mL for IIB, respectively. The infected birds treated with Flagymox and microbial‐based therapies produced a significant reduction (*p* < .05) in both C. *perfringens* and *E. coli* counts when compared to the positive control groups (IB & IIB). The most relevant and significant (*p* < .05) finding was the lowest log10 values of *C*. *perfringens* (1.59 and 1.49 CFU/mL) in the infected Cobb and Arbor Acres groups treated with the microbial‐based products (ID and IID) when compared to other treated and untreated groups (Figure 3).

### 3.6 The effect of antibiotics and microbial‐based therapy on the development of lesions in the jejunum of Cobb and Arbor Acres broilers experimentally infected with *C. perfringens*

**Figure 4:**
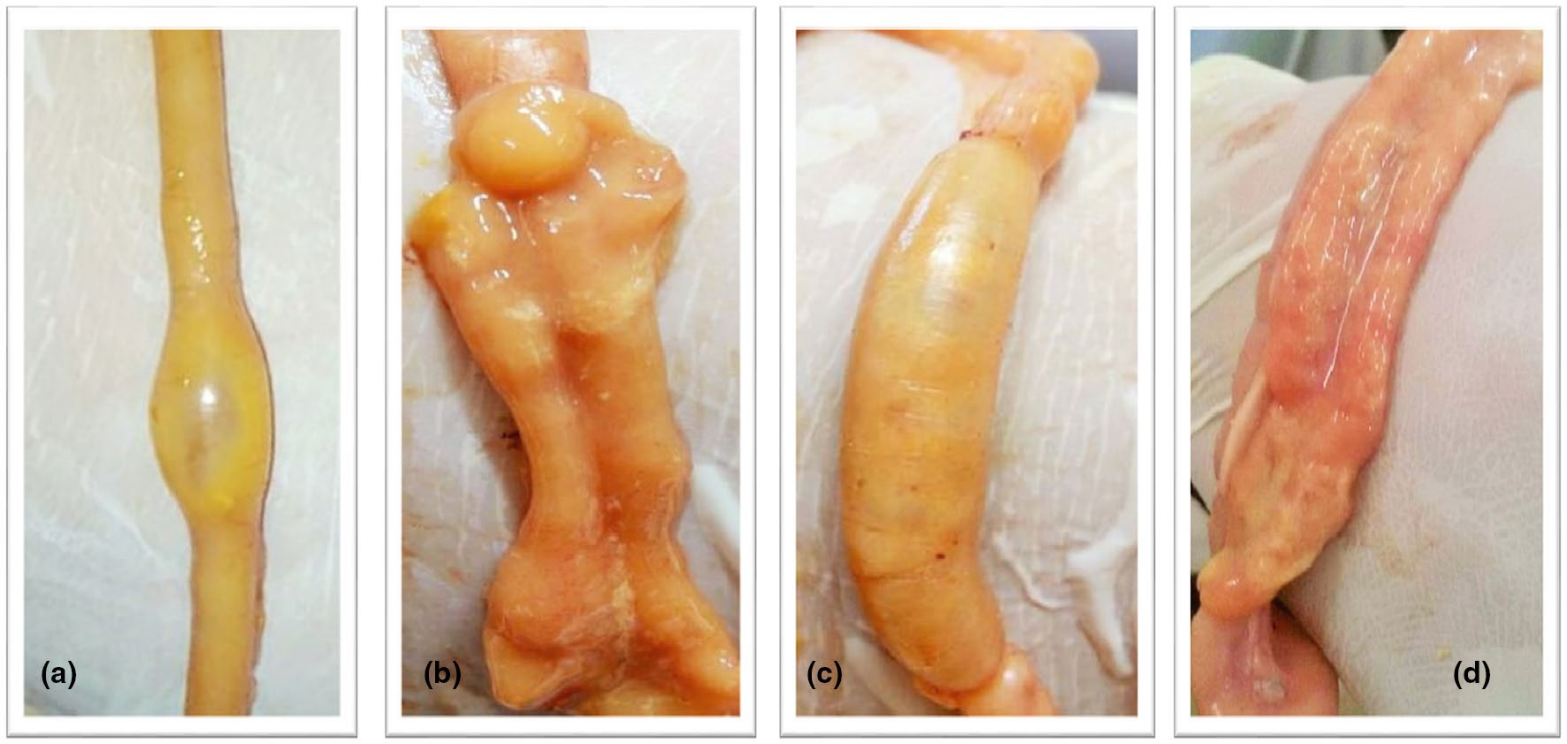
The jejunum of 21‐day old Arbor Acres chickens (A & B) challenged with *C*. *perfringens* show focal necrosis and dilation with a thin wall containing gas (A), hyperemic mucosa with viscus content, fibrin flakes and yellowish‐brown fluids (B). In addition to diffused distension and ballooning observed in the jejunal wall (C) of the *C*. *perfringens* infected Cobb broilers, the necrotic debris and a layer of fibrin were also observed covering the mucosal surface (D)

At necropsy on day 21 of the experiment, the infected Cobb and Arbor Acres groups (IB & IIB) showed macroscopic lesion scores of greater than 2 (Table 4) in the jejunum, which mainly exhibited a hyperemic, thin wall that was filled with gas and yellowish‐brown fluid along with focal or diffused necrotic flakes covering the mucosal surfaces (Figure 4). These macroscopic lesion scores of jejunum showed a clinically positive relationship (a score of 0.177) with its microscopic measurement at the extent of the tissue injury. It is important to note the down‐shift in the macroscopic lesion scores of the treated groups (IC, ID and IIC, IID with the scores of 2, 1 and 1.4, 1.2, respectively) compared to the scores of the positive control groups (IB & IIB with the scores of 2.4 & 2.2, respectively). Consequently, the most efficient (*p* < .05) reduction in the severity of macroscopic lesion scores were achieved in both broiler breeds by the microbial‐based products (groups ID & IID) compared to the other treated groups (IC & IIC).

**Table 4:** Effect of antibiotic and microbial based administration in Cobb and Arbor acres broilers experimentally infected with *Cl*. *perfringens* on the development of Jejunal lesions

Items		Cobb Breed				Arbor acres Breed				Correlation ^≠≠^
Experimental group		IA	IB	IC	ID	IIA	IIB	IIC	IID	
Macroscopic lesion score ^≠^		1.40^bcd^ ± 0.24	2.40^(a‐e)^ ± 0.40	2.00^(a‐e)^ ± 0.32	1.00^d^ ± 0.32	0.80^d^ ± 0.20	2.20^(a‐e)^ ± 0.20	1.40^bcd^ ± 0.24	1.20^cd^ ± 0.20	0.177
Microscopic changes	Necrosis	‐	++++	++	+	‐	+++	+	…	
	Fibrin exudate	‐	++++	++	+	‐	+++	+	…	
	Inflammatory cells	‐	+++	++	+	‐	++	++	…	
	Hyperplasia	‐	++	++	+	‐	+++	+	…	
	Normal villi	+++	+	++	++	++++^√^	+	++	…	
Morphometric variables^•^ (µm)	Villi length	703.74^b^ ± 13.1	176.90^c^ ± 2.2	204.16^c^ ± 7.1	312.13^c^ ± 10.7	1,111.7^(a‐e)^ ± 115.4	198.97^c^ ± 5.6	276.17^c^ ± 4.9	281.82^c^ ± 18.74	‐ 0.657**
	Villi width	63.34^b^ ± 17.3	55.48^bc^ ± 6.5	33.07^c^ ± 3.1	37.75^bc^ ± 3.8	101.74^(a‐e)^ ± 14.5	95.75^(a‐e)^ ± 3.3	44.29^bc^ ± 1.5	50.98^bc^ ± 4.98	‐ 0.128
	Crypt depth	214.81^b^ ± 8.7	46.61^c^ ± 0.6	55.10^c^ ± 4.3	40.96^d^ ± 3.4	296.20^(a‐e)^ ± 14.2	50.43^c^ ± 3.4	56.06^c^ ± 4.3	43.64^c^ ± 1.96	−0.693**
	VL/ CD	3.28^d^ ± 0.1	3.83^d^ ± 0.1	3.73^cd^ ± 0.2	7.72^(a‐e)^ ± 0.6	3.74^d^ ± 0.3	3.99^d^ ± 0.4	4.97^c^ ± 0.3	6.45^b^ ± 0.20	0.387
	Extent of tissue injury	18.79^(a‐e)^ ± 3.8	153.73^(a‐e)^ ± 0.8	115.65^b^ ± 18.4	46.40^c^ ± 3.3	9.20^(a‐e)^ ± 1.5	115.73^b^ ± 3.3	97.85^b^ ± 3.8	32.72^cd^ ± 4.98	1

Note

(^≠≠^): The extent of tissue injury in jejunum was correlated with its macroscopic lesion score and other morphometric variables. **Correlation is significant at the 0.01 level (2‐tailed). Values with – indicate negatively correlation.

^(a‐e)^ Means within the same row with significantly different between treated and untreated groups (*p* ≤ .05). (^≠^) Macroscopic lesion of the examined jejunum (*n* = 5/ group: means ± standard error) were scored as follow: 0 = no gross lesions, normal intestinal appearance; 1 = thin‐walled or friable, gray appearance; 2 = thin‐walled, focal necrosis, gray appearance, small amounts of gas production; 3 = thin walled, sizable patches of necrosis, gas‐filled intestine, small flecks of blood; 4 = severe extensive necrosis, marked hemorrhage, large amounts of gas in intestine. () Microscopic changes of the examined jejunum (*n* = 3/ group) were scored as follow: 0: no lesions; +: mild lesions; ++: moderate lesions; +++: severe lesions and ++++: very severe lesions. (^√^) indicates marked increase of villi length. (^?^) VL/ CD: ratio between Villi length and Crypt depth.

### 3.7 The effect of antibiotics and microbial‐based administration on the microscopic findings of the jejunum in Cobb and Arbor Acres broilers experimentally infected with *C. perfringens*

**Figure 5:**
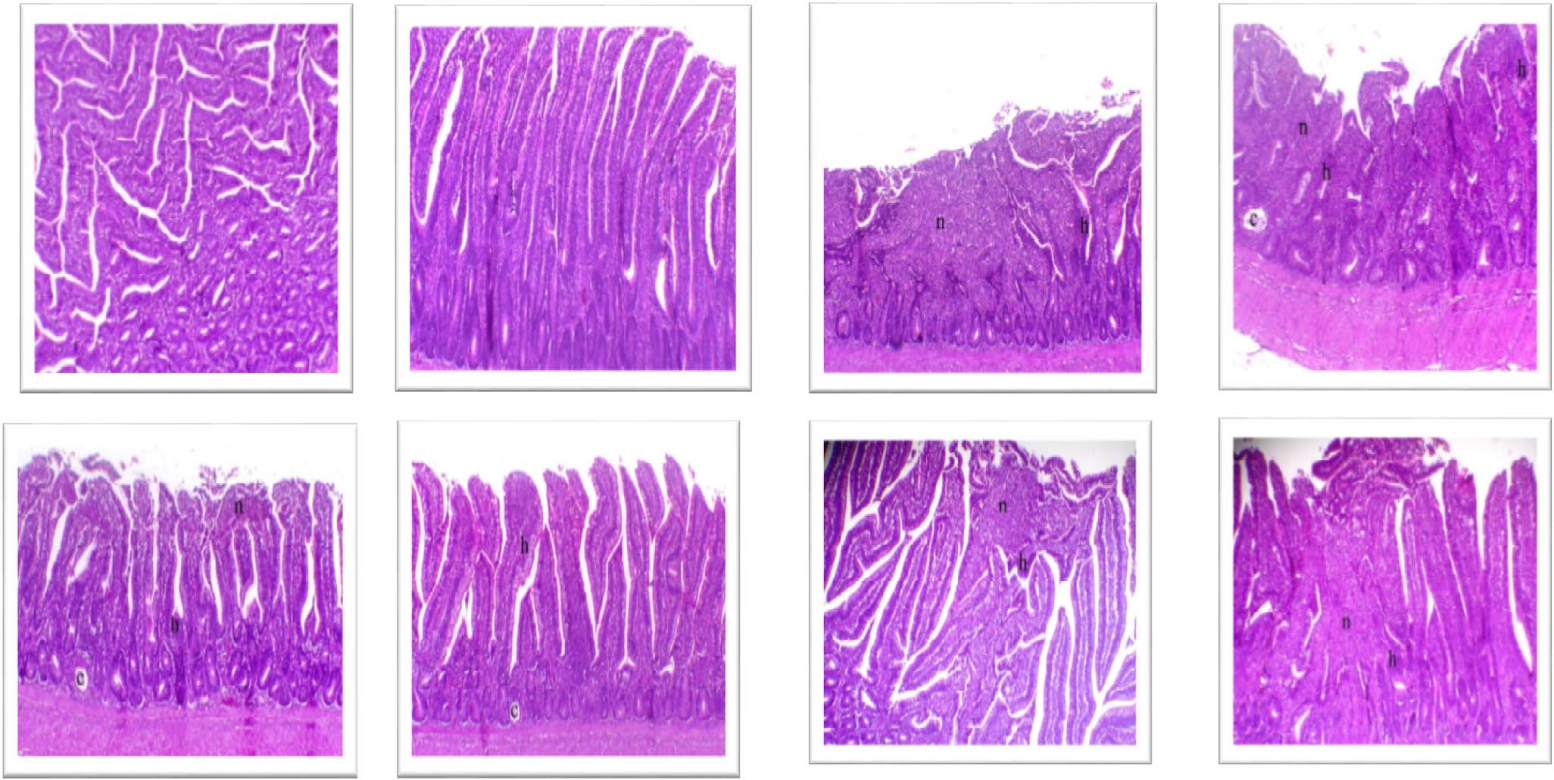
The effect of the microbial or antibiotic administration on the microscopic findings in the jejunum of Cobb and Arbor Acres broilers experimentally infected with *C*. *perfringens*. 1. Jejunum of negative control Cobb broiler showing normal villi, H&E, X50. 2. Jejunum of negative control Arbor Acres broiler showing a marked increase in villi length and branches, H&E, X50. 3. Jejunum of positive control Cobb broiler showing chronic enteritis along the entire mucosal lining (n indicates necrosis and h indicates hyperplastic epithelial lining), H&E, X50. 4. Jejunum of positive control Arbor Acres broiler showing chronic enteritis associated with marked hyperplasia of the epithelial lining (n indicates necrosis, h indicates hyperplastic epithelial lining and c indicates retention cyst), H&E, X50. 5. Jejunum of the *C*. *perfringens* infected Cobb broiler treated with the microbial‐based product showing focal and superficial necrosis with mild hyperplasia of the lining epithelium (n indicates necrosis, h indicates hyperplastic epithelial lining and c indicates retention cyst), H&E, X50. 6. Jejunum of the *C*. *perfringens* infected Cobb broiler treated with the antibiotic‐based product showing marked hyperplasia of the lining epithelium with mostly goblet cells (h indicates hyperplastic epithelial lining and c indicates the retention cyst), H&E, X50. 7. Jejunum of the *C*. *perfringens* infected Arbor Acres broiler treated with the microbial‐based product showing focal enteritis associated mild hyperplasia of the villi lining epithelium (n indicates necrosis and h indicates hyperplastic epithelial lining), H&E, X50. 8. Jejunum of the *C*. *perfringens* infected Arbor Acres broiler treated with the antibiotic‐based product showing moderate enteritis lesions associated with hyperplasia of the villi lining epithelium (n indicates necrosis and h indicates hyperplastic epithelial lining), H&E, X50

Figure 5 shows the differences in the histopathological findings of the jejunum infected with *C*. *perfringens* between antibiotics and microbial therapy treatment groups. Normal tissue architecture with normal intestinal villi was observed in the jejunum of the negative control groups (IA & IIA). In the negative control group (IIA) of Arbor Acres chicks, villi length and branches showed a marked increase. Meanwhile, *C*. *perfringens* infection was effective in inducing severe chronic enteritis along the entire mucosal lining in the jejunum of the infected Cobb and Arbor Acres chicks (IB & IIB) with marked hyperplasia of the epithelial lining and marked infiltration of goblet cells. The severity of these findings was diminished into a moderate feature of enteritis and hyperplasia of the villi lining epithelium in groups IC and IIC, where the antibiotic product was used to treat the infected broilers. Interestingly, focal and superficial enteritis with mild hyperplasia of the villi lining epithelium was documented in the jejunal tissue of the *C*. *perfringens* infected Cobb, and Arbor Acres broilers (ID & IID) treated with the microbial product.

### 3.8 The effects of the antibiotics and microbial therapy on the jejunal histomorphometric measurements of the *C. perfringens* infected Cobb and Arbor Acres broiler chicks

The jejunal histomorphometric measurements (villus length, villus width, crypt depth, the ratio between villus length and crypt depth (VH: CD ratio) and the extent of tissue injury) are presented in Table 4. In all the treated and untreated groups, a significant (*p* < .01) negative correlation was documented between the extent of tissue injury and the villus length or crypt depth. Consequently, the negative adverse effects of the C. perfringens infection on the morphology of the jejunum were presented as a significant (*p* < .05) decrease in the values of the villus length and crypt depth along with a significant (*p* < .05) increase in the extent of tissue injury when compared to those of the negative control groups (IA and IIA). Interestingly, a significant decrease (*p* < .05) was observed in the extent of the jejunal tissue injury in the antibiotics, and microbial treatment group of the infected Cobb chicks (IC & ID) and in the microbial therapy group of the infected Arbor Acres chicks (IID) when compared to the positive control groups IB and IIB, respectively. Moreover, the ratio is another important indicator of intestinal recovery and health. No significant differences were observed between the positive control groups (IB & IIB) and the negative control groups (IA & IIA), respectively. In this study, the highly significant VH: CD ratio was recorded in the group ID, IID and IIC when compared to the positive control groups of both the breeds (Table 4).

### 3.9 The effect of antibiotics and microbial‐based administration on mRNA expression of jejunal IL6 and IL8 in Cobb and Arbor Acres broilers experimentally infected with *C. perfringens*

**Figure 6:**
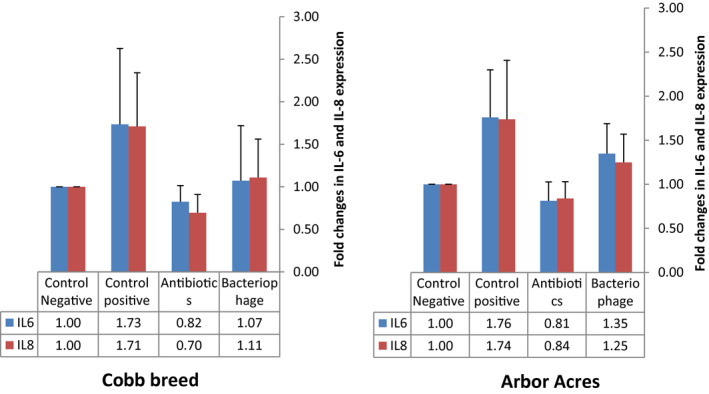
The effect of antibiotics and microbial‐based administration on mRNA expression of the jejunal IL6 and IL8 in Cobb and Arbor Acres broilers experimentally infected with *C*. *perfringens*

Changes in the intestinal (jejunal) IL6 and IL8 mRNA expression are presented in Figure 6. The expression of IL6 and IL8 showed non‐significant (*p* < .05) changes in the positive control groups (IB & IIB) compared to other groups with both Cobb broiler and Arbor Acres breeds. The antibiotic‐treated groups (IC & IIC) showed up‐regulation of IL6 and IL8 mRNA numerically compared to the negative control groups (IA & IIA) either in Arbor Acres and Cobb broiler breeds. In the groups treated with Flagymox and microbial‐based products, a numerical non‐significant (*p* < .05) normalization was observed in IL6 and IL8 expression compared to the groups challenged with *C*. *perfringens* (Figure 6).

### 3.10 The effect of antibiotics and microbial‐based administration on carcass traits of experimentally infected Cobb and Arbor Acres broilers with *C. perfringens*

Table 5 shows that the groups treated with the microbial‐based product (ID & IID) in both Cobb and Arbor Acres breed were superior in dressing percentage (75.16 and 77.06%, respectively) compared to the other groups. Among different groups, the intestine percentage showed a significant difference, with the highest value (6.09%) being recorded in the positive control group of Arbor Acres breed (IIB). In general, the positive group (IB & IIB) in each breed had a higher value of intestine percentage compared to the other groups of the same breed.

**Table 5:** Effect of antibiotic and microbial based administration in Cobb and Arbor acres broilers experimentally infected with *Cl*. *perfringens* on carcass trait

Items	Cobb Breed					Arbor acres Breed				
	IA	IB	IC	ID	Overall mean	IIA	IIB	IIC	IID	Overall mean
Dressing %	72.96^b^ ± 0.32	72.07^b^ ± 0.41	74.73^ab^ ± 0.83	75.16^ab^ ± 1.12	73.67^A^ ± 0.46	74.92^ab^ ± 0.88	72.70^b^ ± 0.63	73.43^b^ ± 1.98	77.06^a^ ± 1.21	74.74^A^ ± 0.71
Liver %	2.66**^ab^** ± 0.28	2.89**^ab^** ± 0.41	2.54**^ab^** ± 0.16	2.16**^b^** ± 0.12	2.57**^A^** ± 0.14	2.46**^ab^** ± 0.03	2.98**^a^** ± 0.21	3.17**^a^** ± 0.22	3.09**^a^** ± 0.28	2.90**^A^** ± 0.12
Heart %	0.49**^b^** ± 0.02	0.66**^a^** ± 0.05	0.52**^ab^** ± 0.04	0.57**^ab^** ± 0.02	0.55**^A^** ± 0.02	0.49**^b^** ± 0.03	0.56**^ab^** ± 0.06	0.52**^ab^** ± 0.04	0.64**^ab^** ± 0.08	0.55**^A^** ± 0.03
Gizzard %	1.72**^a^** ± 0.13	1.80**^a^** ± 0.20	2.02**^a^** ± 0.35	1.89**^a^** ± 0.07	1.85**^A^** ± 0.09	1.50**^a^** ± 0.17	1.81**^a^** ± 0.04	1.72**^a^** ± 0.09	1.67**^a^** ± 0.04	1.66**^A^** ± 0.06
Intestine %	5.10**^ab^** ± 0.34	5.25**^ab^** ± 0.33	5.12**^ab^** ± 0.59	4.57**^b^** ± 0.02	5.02**^A^** ± 0.18	5.25**^ab^** ± 0.47	6.09**^a^** ± 0.29	5.53**^ab^** ± 0.33	4.94**^ab^** ± 0.20	5.40**^A^** ± 0.19
Abdominal fat %	1.57**^a^** ± 0.23	0.68**^bc^** ± 0.09	1.16**^abc^** ± 0.36	0.45**^c^** ± 0.04	1.01**^A^** ± 0.16	1.26**^ab^** ± 0.31	0.52**^c^** ± 0.06	0.97**^abc^** ± 0.18	0.79**^bc^** ± 0.09	0.86**^A^** ± 0.13
Gizzard fat%	0.14**^b^** ± 0.01	0.88**^a^** ± 0.07	0.84**^a^** ± 0.17	0.80**^a^** ± 0.30	0.62**^A^** ± 0.12	0.13**^b^** ± 0.01	0.74**^a^** ± 0.21	0.52**^ab^** ± 0.11	0.29**^b^** ± 0.05	0.39**^A^** ± 0.08
Spleen %	0.17**^a^** ± 0.03	0.19**^a^** ± 0.04	0.12**^a^** ± 0.01	0.15**^a^** ± 0.01	0.16**^A^** ± 0.02	0.17**^a^** ± 0.00	0.21**^a^** ± 0.04	0.17**^a^** ± 0.04	0.19**^a^** ± 0.01	0.18**^A^** ± 0.01
Bursa %	0.15**^a^** ± 0.03	0.11**^ab^** ± 0.01	0.11**^ab^** ± 0.03	0.06**^b^** ± 0.00	0.11**^A^** ± 0.01	0.06**^b^** ± 0.01	0.11**^ab^** ± 0.00	0.07**^b^** ± 0.00	0.08**^b^** ± 0.02	0.08**^B^** ± 0.01
Thymus %	0.38**^a^** ± 0.06	0.24**^a^** ± 0.02	0.37**^a^** ± 0.10	0.39**^a^** ± 0.03	0.36**^A^** ± 0.03	0.26**^a^** ± 0.03	0.33**^a^** ± 0.06	0.32**^a^** ± 0.07	0.36**^a^** ± 0.05	0.32**^A^** ± 0.02

Note

Small letters are significantly different between treated and untreated groups (*p* ≤ .05), while Capital letters significantly differ among different breeds.

The lowest value of the abdominal fat was found in the group treated with the microbial‐based product (0.45%) in Cobb breed. For the spleen and thymus, non‐significant differences were observed between different groups. A non‐significant breed effect was reported on dressing, intestine, abdominal fat, gizzard fat, spleen and thymus percentages. In contrast, there was a significant effect of the breed on bursa percentage, where Cobb breed (0.11%) exceeded the Arbor Acres breed (0.08).

### 3.11 The effect of antibiotics and microbial‐based administration on different cost patterns (total veterinary management, the total variable cost, total fixed cost and total cost) of Cobb and Arbor Acres broilers experimentally infected with *C. perfringens*

Table 6 shows that **the** feed cost was non‐significant between different treatment groups, with treatment cost being the highest in the antibiotics treated group (LE 0.86) and lower in the microbial‐based treatment group (LE 0.35). On the other side, the total variable cost (TVC) and total cost (TC) showed significant differences in the antibiotics treated groups for both the breeds (IC & IIC, respectively), demonstrating higher values of TVC (LE 36.43 and 36.14) and TC (LE 38.04 and 37.75) while the lowest values (LE 34.06 and 35.67 for TVC and TC, respectively) were found in the positive group of Cobb breed (IB).

**Table 6:** Effect antibiotic and microbial based administration in Cobb and Arbor acres broilers experimentally infected with *Cl*. *perfringens* on different cost patterns (total veterinary management, total variable cost, total fixed cost and total cost)

Items	Cobb Breed					Arbor acres Breed				
	IA	IB	IC	ID	Overall mean	IIA	IIB	IIC	IID	Overall mean
Chick price	6.3	6.3	6.3	6.3	6.3	6.3	6.3	6.3	6.3	6.3
Litter cost	0.8	0.8	0.8	0.8	0.8	0.8	0.8	0.8	0.8	0.8
Vaccine	0.4	0.4	0.4	0.4	0.4	0.4	0.4	0.4	0.4	0.4
Disinfectant	0.2	0.2	0.2	0.2	0.2	0.2	0.2	0.2	0.2	0.2
Drug	0	0	0.86	0.35	0.3	0	0	0.86	0.35	0.3
TVM	0.6	0.6	1.46	0.95	0.9	0.6	0.6	1.46	0.95	0.9
Feed cost	26.56**^a^** ± 0.84	25.26**^a^** ± 0.22	26.77**^a^** ± 0.26	26.10**^a^** ± 0.40	26.17**^A^** ± 0.27	25.74**^a^** ± 0.76	26.39**^a^** ± 0.42	26.48**^a^** ± 0.51	25.63**^a^** ± 1.00	26.06**^A^** ± 0.32
Water& electricity	0.1	0.1	0.1	0.1	0.1	0.1	0.1	0.1	0.1	0.1
Labor	1	1	1	1	1	1	1	1	1	1
TVC	35.36**^ab^** ± 0.84	34.06**^b^** ± 0.22	36.43**^a^** ± 0.26	35.25**^ab^** ± 0.40	35.27**^A^** ± 0.33	34.54**^ab^** ± 0.76	35.19**^ab^** ± 0.42	36.14**^a^** ± 0.51	34.78**^ab^** ± 1.00	35.16**^A^** ± 0.35
TFC	1.61	1.61	1.61	1.61	1.61	1.61	1.61	1.61	1.61	1.61
TC	36.97**^ab^** ± 0.84	35.67**^b^** ± 0.22	38.04**^a^** ± 0.26	36.86**^ab^** ± 0.40	36.88**^A^** ± 0.33	36.15**^ab^** ± 0.76	36.80**^ab^** ± 0.42	37.75**^a^** ± 0.51	36.39**^ab^** ± 1.00	36.77**^A^** ± 0.35
Total losses from mortality	24.75	98.42	0.00	0.00	30.79	24.14	73.61	17.52	0.00	28.82

Note

Small letters are significantly different between treated and untreated groups (*p* ≤ .05), while Capital litters significantly differ among breeds. TVM (total veterinary management), TVC (total variable cost), TFC (total fixed cost) and TC (total cost). TVM (=drug + vaccine+disinfectant). TVC( = chick price + feed cost + tvm +water and electricity + labour+litter cost)

Due to the post‐infection mortality, positive groups of both the breeds (IB & IIB) suffered high losses (LE 98.42 and 73.61 for Cobb and Arbor Acres breed, respectively) compared to the other groups. However, no losses were observed in the treatment groups of the microbial‐based product and antibiotics in both the breeds (ID & IID) and Cobb breed (IC), respectively. Thus, treatment either with antibiotics or with microbial‐based products resulted in better results.

### 3.12 The effect of antibiotics and microbial‐based administration on different return parameters (Selling of litter, Selling of broiler chicken, total returns and net profit) in Cobb and Arbor Acres broilers experimentally infected with *C. perfringens*

The results presented in Table 7 showed that returns from selling broiler chicken, and the total returns (TR) were higher in challenged and treated groups compared to the positive control group of both breeds. The highest value of returns from selling broiler chicken along with the total returns was recorded in the antibiotics treated group of Cobb breed and Arbor Acres (IC & IIC) (returns from selling broiler chicken were LE 44.51 and 42.67, and the TR were 44.91 and 43.07 LE for IC & IIC, respectively) and the lowest value was recorded in the positive group of Cobb breed (the returns from selling broiler chicken was LE 38.62 and the TR was 39.02 LE, respectively).

**Table 7:** Effect of antibiotic and microbial based administration in Cobb and Arbor acres broilers experimentally infected with *Cl*. *perfringens* on different return parameters (Selling of litter, selling of broiler chicken, total return and net profit)

Items	Cobb Breed					Arbor acres Breed				
	IA	IB	IC	ID	Overall mean	IIA	IIB	IIC	IID	Overall mean
Selling of litter.	0.4	0.4	0.4	0.4	0.4	0.4	0.4	0.4	0.4	0.4
Selling of broiler chicken	40.22 **^a^** ± 2.35	38.62 **^a^** ± 2.20	44.51 **^a^** ± 3.75	41.60 **^a^** ± 1.63	41.23**^A^** ± 1.29	42.57 **^a^** ± 1.20	41.85 **^a^** ± 0.27	42.67 **^a^** ± 1.82	42.45 **^a^** ± 2.94	42.39**^A^** ± 0.79
Total return (TR)	40.62 **^a^** ± 2.35	39.02 **^a^** ± 2.20	44.91 **^a^** ± 3.75	42.00 **^a^** ± 1.63	41.63**^A^** ± 1.29	42.97 **^a^** ± 1.20	42.25 **^a^** ± 0.27	43.07 **^a^** ± 1.82	42.85 **^a^** ± 2.94	42.79**^A^** ± 0.79
Net profit	3.65 **^a^** ± 1.73	3.35 **^a^** ± 2.00	6.87 **^a^** ± 3.84	5.14 **^a^** ± 2.00	4.75**^A^** ± 1.16	6.82 **^a^** ± 0.96	5.45 **^a^** ± 0.51	5.32 **^a^** ± 1.35	6.46 **^a^** ± 2.01	6.01**^A^** ± 0.60

Note

Selling of broiler = final body weight per gram × market price per gram (Market price/g = 0.026 LE).TR = selling of broiler + selling of litter.

Small letters are significantly different between treated and untreated groups (*p* ≤ .05), while Capital litters significantly differ among breeds.

Net profit was higher in all the treated groups of Cobb breed (LE 6.87 and 5.14 for IC & ID) compared to the positive group (IB), while the group with Arbor Acres breed treated with the microbial based product (IID) showed a higher value (LE 6.46) compared to the positive control group (IIB) (LE 5.45). Considering the breed effect, the Arbor Acres breed showed a higher net profit compared to the Cobb breed (LE 6.01 and 4.75, respectively).

### 3.13 The effect of antibiotics and microbial‐based administration on economic efficiency and relative economic efficiency of Cobb and Arbor Acres broilers experimentally infected with *C. perfringens*

**Figure 7:**
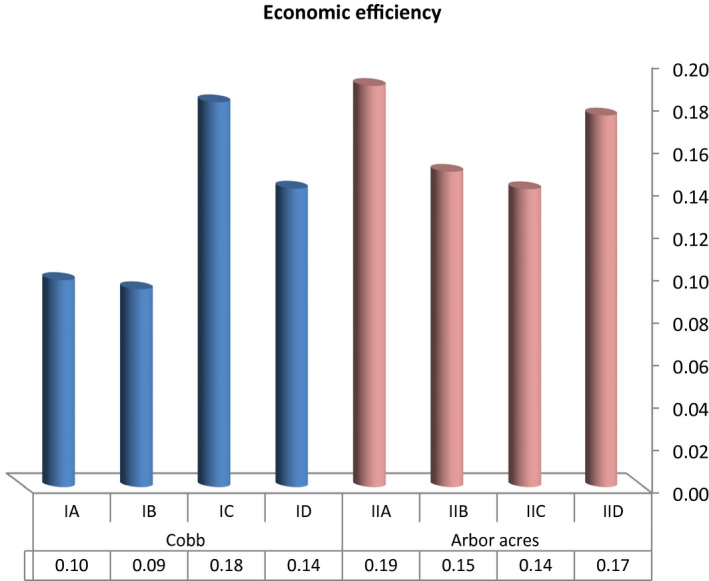
The effect of antibiotics and microbial‐based administration on the economic efficiency of Cobb and Arbor Acres broilers experimentally infected with *C*. *perfringens*

**Figure 8:**
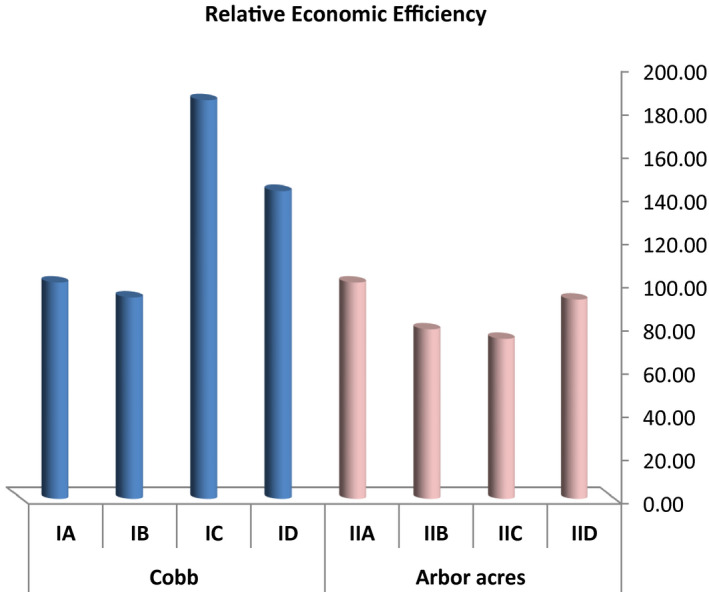
The effect of antibiotics and microbial‐based administration on the relative economic efficiency of Cobb and Arbor Acres broilers experimentally infected with *C*. *perfringens*

Economic efficiency and relative economic efficiency (Figures 7 and 8) showed non‐significant differences (*p* > .05) between both the breeds. In the groups of Cobb breed treated with antibiotics and microbial based products (IC & ID), both economic efficiency (0.18, 0.14, and 0.09 for IC, ID, and IB, respectively) and relative economic efficiency (184.40 and 142.38, respectively) were superior compared to the positive control group (93.14 for IB) while the groups of Arbor Acres treated with the microbial‐based product (IID) showed a higher value of economic efficiency and relative efficiency (0.17 and 92.14, respectively) compared to the positive control (IIB) (0.15 and 78.29 for economic efficiency and relative efficiency, respectively).

## 4 DISCUSSION

Intestinal health is still one of the most important subjects in poultry production because the impact of digestion and nutrient absorption can harm the feed efficiency, increase the susceptibility to diseases, and eventually cause economic losses. Among the intestinal diseases in poultry, NE is economically more important as it impairs production and economic performance in broiler chicken (Caly et al., 2015). This disease is being controlled by antimicrobials for many years in the poultry industry. Recently, microbial‐based products are being used as alternatives in disease prevention that also act as supplements for growth enhancement, aiming to reduce the use of antibiotics in the broiler industry (Seal et al., 2018). Thus, there is an urgent need to explore the full practical potential of microbial therapy. In this context, the present study explored microbial‐based product as an alternative for the antibiotics in the treatment of NE in two different broiler breeds, where its effects were investigated on broiler production and economic performance, microbial counts, intestinal macroscopic and microscopic lesion with its histomorphology, carcass composition and gene expression.

Current findings confirmed that the antibiotics treated groups in both Cobb and Arbor Acres breeds achieved an increase in BW, FI and dressing percentage compared to the positive control group in the third week. Similar findings by Abudabos et al. (Abudabos et al., 2018; Abudabos & Yehia, 2013) reported that the *C*. *perfringens* challenged group treated by avilamycin had higher body weight, dressing percentage and feed intake at the third week. Moreover, other studies (Elbayoumi et al., 2014) have reported that the groups challenged with C. perfringens were the most affected in their average weekly body weight and FCR during the first week of post‐infection, while the groups treated with the antibiotics showed improved body weight and FCR. The results of TWG from zero to fifth week were in line with those obtained by Miller et al., 2010, who stated that the greater weight gains were recorded from 0 to 35‐day period in all the bird groups treated with phages compared to the challenged control.

The results for mortality percentage and the counts of *C*. *perfringens* and *E*. *coli* in the jejunum were found to be similar in other studies as well (Jayaraman et al., 2013; McReynolds et al., 2004, 2009), where a significant increase in the mortality and the intestinal microbial quantities were observed in all the experimental chicks. Interestingly, both antibiotics and microbial products had a positive effect on the mortality rate and quantities of *C*. *perfringens* and *E*.*coli* in the jejunum. These findings were similar to other reports, where *C*. *perfringens* infected broilers were treated with the microbial product by direct feed via the drinking water (McReynolds et al., 2009), or supplemented with six bacteriophages (Miller et al., 2010) or treated with probiotics or antibiotics (Hussein, Ahmed, Abudabos, Aljumaah, et al., 2020; Jayaraman et al., 2013). Moreover, the synergistic effect of a combined treatment that includes phages and bacteriocins to control *C*. *perfringens* showed a significant reduction in the bacterial population (Heo et al., 2018). Hence, a significant reduction in the mortality rate and intestinal microbes may be due to the inhibitory effects of the antibiotics and microbes towards the enteric microorganisms (Chichlowski et al., 2007).

In the present study, the pathological findings in the jejunum of the infected Cobb and Arbor Acres groups were characterized by the development of NE lesions at day 21 post*‐*hatch. Moreover, the jejunal morphology measurements demonstrated a significant decrease in the villus length and crypt depth, which negatively correlated with the significant increase in the extent of tissue injury compared to the negative control groups of Cobb and Arbor Acres breed. These findings coincide with the previously reported papers that have illustrated the negative effects of *C*. *perfringens* and its toxins on the sloughing and chronic inflammation of the intestine, which is also associated with high intestinal lesion scores and shortening of the villus length. This directly affects the nutrient absorption while indirectly affecting the performance of the birds (Gholamiandehkordi et al., 2007; Hussein, Ahmed, Abudabos, Aljumaah, et al., 2020; M’Sadeq et al., 2015; McReynolds et al., 2009; Star et al., 2009; Xu et al., 2003).

The severity of the pathological findings and the histomorphology changes in the jejunum of the infected Cobb and Arbor Acres groups were reduced because of the antibiotics or microbial‐based treatments. These findings partially concurred with the findings of the other researchers (Calik et al., 2019; Hussein, Ahmed, Abudabos, Aljumaah, et al., 2020; Jayaraman et al., 2013; M’Sadeq et al., 2015) who have reported the ameliorative effects of different feed supplementations such as antibiotics, phytobiotics, prebiotics or probiotics in developing the NE lesions in the intestine of the broiler chickens. This effect may be attributed to the activity of the used drug that reduces the irritation caused by *C*. *perfringens* while preserving the integrity of the intestinal epithelial layer. Antibiotics and bacteriophages directly affect the pathogenic bacteria by causing their death and preventing further bacterial growth. Moreover, bacteriophages and probiotics produce anticlostridial factors that maintain favourable ecological homeostasis for the intestinal microbes to promote the development of gut immunity and histomorphology (Caly et al., 2015; Khalique et al., 2020; Li et al., 2018; Seal et al., 2018; Wernicki et al., 2017; Whelan et al., 2019; Zimmer et al., 2002). The results obtained in this study, including no mortalities, low C. perfringens log10 values, mild macroscopic and microscopic NE lesions were consistent with those reported in controlling the *C*. *perfringens* infection in the broilers using probiotics (Calik et al., 2019; Hussein, Ahmed, Abudabos, Aljumaah, et al., 2020; Jayaraman et al., 2013; M’Sadeq et al., 2015; McReynolds et al., 2009; Wang et al., 2017) and bacteriophages (Miller et al., 2010). These results occur more frequently than expected because of the synergistic ameliorative effects of the probiotic and bacteriophages used along with the drugs against the *C*. *perfringens* infection in the broilers.

IL‐6 is a multifunctional cytokine with a pro‐inflammatory activity that induces acute‐phase protein synthesis as well as aids the adaptive immune response (Kaiser et al., 2000). IL‐8 is a chemokine produced by the macrophages that functions primarily as a chemoattractant and plays an important role in inflammation (Shahzad et al., 2010). Here, *C*. *perfringens* challenged groups showed a numerical up‐regulation in the expression of IL6 and IL8 due to the inflammation induced by the bacterial infection. It is shown that necrotic enteritis in broiler chicken results in increased expression of IL8, while the expression of IL6 is not altered (Park et al., 2008). Challenging with *C*. *perfringens* up‐regulates the mRNA expression of interleukins (IL6 and IL8) in primary intestinal epithelial cells (Guo et al., 2015). In our study, the group treated with antibiotics showed a non‐significant down‐regulation in IL6 and IL8 compared to the negative control group either in Arbor Acres or Cobb broiler breeds. The decrease in the expression of the interleukins clarifies the role of antibiotics in treating intestinal inflammation. Both the breeds treated with the microbial‐based product showed an up‐regulation in IL6 and IL8 expression compared to the antibiotics treated groups, which may be due to the activation of the immune system since the microbial‐based product increases the phagocytosis of bacteria by macrophages (Adhikari et al., 2017).

In this study, no significant differences were observed between non‐infected and infected birds in the carcass quality, including dressing, bursa, spleen and gizzard percentage, which also coincided with other research findings (Hussein, Ahmed, Abudabos, Suliman, et al., 2020). Contrastingly, some studies (Elmenawey et al., 2019) have recorded a significant increase in the above‐mentioned carcass qualities. In general, the positive control group of each breed has a higher value of intestine percentage compared to the other groups of the same breed, which may reflect the degree of inflammation in the jejunum along with the accumulation of the exudates in the intestines of the infected groups (Abudabos et al., 2016; Alzawqari et al., 2016).

The microbial‐based treatment was less expensive (LE 0.35) compared to the antibiotics treatment. Hence, the microbial‐based treatment offered an inexpensive treatment. The total returns (TR) were higher in all the treated groups compared to the positive control group of both the breeds. The highest value of TR was found in the antibiotics treated group of Cobb breed, whereas the lowest value was found in the positive group of Cobb breed. Net profit was higher in all the treated groups (IC, ID) compared to the positive control group of Cobb breed. Our results can explain the reported reduction in weight gain, poor performance and increased costs for the poultry producers (Abudabos & Yehia, 2013; Wade et al., 2015). Similarly, necrotic enteritis was considered to reduce the body weight of the infected birds compared to the healthy birds (Skinner et al., 2010).

Regarding the total losses due to mortality, the positive group recorded the highest losses among different groups of both breeds, whereas no losses were found in the groups treated with the microbial‐based product and antibiotics in both the breeds and Cobb breed, respectively. These results were in agreement with the other studies (Geier et al., 2010; Hussein, Ahmed, Abudabos, Aljumaah, et al., 2020; Miller et al., 2010).

Although a non‐significant difference was observed in the economic efficiency and relative economic efficiency, the microbial‐based product and antibiotics succeeded in improving the economic efficiency (Choct & Kocher, 2008; Skinner et al., 2010; Wade et al., 2015). It was found that necrotic enteritis reduced the body weight of the birds and increased the cost of production, which could be controlled using antibiotics (Williams, 2005). Also, using alternative strategies to control NE could limit the economic impact of the disease (Dahiya et al., 2007; M’Sadeq et al., 2015; Miller et al., 2010). Moreover, the addition of probiotics had a positive effect on the performance of broiler chickens infected with NE (Hussein, Ahmed, Abudabos, Aljumaah, et al., 2020), which was also reflected in economic efficiency.

## 5 CONCLUSION

The poultry constitutes 8% of the reported food‐borne disease outbreaks, and since the microbial‐based treatment prevents foodborne pathogens in the broiler food chain, it confirms the hygienic importance of using the microbial‐based treatment in controlling *C*. *perfringens*. Also, the bio‐control of *C*. *perfringens* infection using the microbial‐based product is considered a natural, hygienic and relatively inexpensive method to control poultry health problems. The microbial‐based product also protects chickens from being biohazardous because of *C*. *perfringens* infection.

## CONFLICT OF INTEREST

Authors declare that there is no conflict of interest for current data.

## AUTHORS CONTRIBUTIONS

EAS, LSM, AEA, *SSE*: conception and design, acquisition of data, writing and drafting of manuscript. MMS: drafting the article or revising it critically for important intellectual content. EAS, LSM, *SSE*, AEA, MMS: approval of the version to be submitted and any revised version.

## FUNDING INFORMATION

This study was supported by Taif University Researchers Supporting Project number (TURSP‐2020/09), Taif University, Taif, Saudi Arabia.

## ETHICAL APPROVAL

This study was approved by Institutional Animals Care and Use committee Research Ethics Board, Faculty of Veterinary Medicine, Benha University, under ethical number BUFVTM 01–09–20.

### PEER REVIEW

The peer review history for this article is available at https://publons.com/publon/10.1002/vms3.412.
